# Editorial: Case reports in autism: 2023

**DOI:** 10.3389/fpsyt.2025.1563977

**Published:** 2025-02-17

**Authors:** Fengyu Zhang, Marco Colizzi

**Affiliations:** ^1^ Global Clinical and Translational Research Institute, Bethesda, MD, United States; ^2^ Unit of Psychiatry and Eating Disorders, Department of Medicine (DMED), University of Udine, Udine, Italy

**Keywords:** autism, cannabinoid system, digital medicine, autism co-morbidity, cognitive behavioral therapy

Autism is a neurodevelopmental disorder that appears early in life and may continue into adulthood or throughout life. As an emerging condition, autism has been shown to have high heterogeneity in its etiology, clinical manifestations, and complex co-morbidities which may complicate the process of making appropriate diagnoses and delivering effective interventions. While some antipsychotics (risperidone and aripiprazole) have been approved for the treatment of aggression and irritability in individuals with autism, there has been no approved pharmacological medication for the treatment of autism (1). People have been actively participating in exploring non-pharmacological therapies to mitigate the clinical symptoms, hoping to support the neurodevelopmental trajectory effectively and timely.

This Research Topic publishes eight reports as part of the “*Case Report in Autism*” series (2). Three reports were on psychiatric co-occurrences with autism and five reports explored novel non-pharmacological interventions on core symptoms or co-morbidities.

Autism has a strong sex bias in diagnosis and girls tend to receive a delayed diagnosis than males due to manifesting symptoms that may not meet existing diagnostic criteria or camouflaging (3). Passarini et al. described clinical and interventional trajectories of a 14-year-old girl who received a late diagnosis of autism with the initial appearance of avoid-restrict food intake disorder (ARFID) and unspecified depression disorder, but normal cognitive functioning. The report provides additional knowledge on the phenotype of female autism. It indicates how psychiatric co-occurrences and normal mental functioning in females could delay the diagnosis and affect timely intervention. *Hikikomori* is described as a clinical condition of pathological social withdrawal or social isolation that lasts at least more than six months and leads to significant impairment in functioning (4). It was initially defined in the Japanese population as a culture-bound syndrome and now has gained recognition in Western society (5) and may connect with several psychiatric disorders including internet addiction and gaming disorder (6). Carpita et al. reported two adult males with *hikikomori*, and both had displayed autistic traits since early infancy. However, they did not turn to medical services until developing severe *hikikomori*-like syndrome that required them to take action. Also, both reported having experienced internet gaming during the social withdrawal before. These suggest that autism may lead to the development of psychiatric co-morbidity with *hikikomori* and internet gaming disorder if not diagnosed and intervened early. After pharmacological treatment, the two cases had improvement in anxiety and mood conditions and reduced social withdrawal and computer gaming time.

The 20q11.21 duplication is known to cause some developmental features (e.g., metopic ridging/trigonocephaly, epicanthal folds, short hands), and developmental delay or syndromic disorders such as Borhing-Opitz syndrome (7). Simoncini et al. reported a 24-year-old woman with a 350kb microduplication at 20q11.21, which segregates in her father and siblings including twin brothers and two sisters, all of whom have presented either subthreshold or full ASD along with other neurodevelopmental or psychiatric disorders such as bipolar, intellectual disability. This genomic region is known to harbor six protein-coding genes.

Immune dysregulation has been widely associated with clinical symptoms or co-morbidity of autism (8). Meanwhile, the endocannabinoid system has recently received great attention for its pharmacological functions of immunoregulation and neuromodulation in multiple physiological and cognitive processes, and two endocannabinoid receptors are expressed in both the central and peripheral nervous system (9). Bortoletto et al. conducted a monotherapy of ultra-micronized-palmitoylethanolamide in two autistic adult females with different levels of severity. One patient showed improvement in depressive symptoms and social engagement, a trend toward reduced inflammatory response, and enhanced endocannabinoid signaling at a 12-week follow-up. In contrast, the severe patient with moderate intellectual disability was observed with a largely stable psychosocial functioning and no change in immune response and endocannabinoid levels, but suddenly became deteriorating and required antipsychotic medications after 12 weeks; no side effects were observed in both cases.

The next four reports focused on non-pharmacological interventions in autism. Maggio et al. presented a nine-year-old girl with autism whose food consumption was primarily restricted to liquid or semi-liquid and who had little success in a private treatment of food selectivity. Following a multicomponent intervention protocol and an AB-type experimental design, they introduced semi-solid and new foods and observed a noted improvement in food acceptance with little behavioral problems at mealtime.

Digital tools have recently gained popularity for measurement and intervention in individuals with autism (10). Receptive language skill involving auditory-visual conditional discrimination is the ability to understand and respond to spoken language, which poses a significant challenge in individuals with autism. The Green’s conditional-only approach has been considered a more reliable and efficient teaching method for autism (11). Minutoli et al. compared the stimulus presentations using flashcards and digital (tablet) platforms in a six-year-old boy with autism and found that the child responded to flashcards faster and more accurately than Tablet slide presentations before reaching a non-error trial. While this is consistent with a previous case study of three participants in comparing three different teaching methods (12), a formal study is needed before a conclusion is drawn. In contrast, Mavritsakis reported that a 15-year-old male with autism had noted improvements in communication and social interaction after the effective introduction of augmentative and alternative communication devices, starting from a letter board and subsequently transitioning to digital platforms such as iPad applications.

Sensory atypicality is prevalent in autism and sensory reactivity and autistic traits are highly heritable and moderately correlated (13). Hyperacusis in individuals with autism often causes emotional distress in their daily lives (14), and there are no proven effective interventions available. Carson et al. introduced cognitive behavioral therapy with exposure, proven effective in reducing anxiety and distress, to treat hyperacusis in an 11-year-old boy with a pervasive developmental disorder not otherwise specified (PDD-NOS), a measurable improvement in auditory sensory domains and tolerance for some noise encountered during his daily life.

In summary, this Research Topic reported psychiatric co-occurrence with autism, some of which could delay the diagnosis of autism and require timely intervention; several novel intervention strategies including digital tools were tried to mitigate the core symptoms and co-morbidity of autism.
